# Selective Cytotoxicity of Sodium Enone Salts Through Mitochondrial Dysfunction and Cell Cycle Arrest in Human Cancer Cells

**DOI:** 10.3390/molecules31071141

**Published:** 2026-03-30

**Authors:** Nikola Mirković, Marina Mitrović, Mirela Jevtić, Katarina Pantić, Petar Čanović, Ivana Nikolić, Stefan Jakovljević, Marina Kostić, Jelena Živić, Jelena Nešić, Nenad Zornić, Stevan Erić, Jovana Muškinja, Marija Šorak, Marija Anđelković

**Affiliations:** 1 Department of Surgery, Faculty of Medical Sciences, University of Kragujevac, 34000 Kragujevac, Serbia; nikola.mirkovic@fmn.kg.ac.rs (N.M.); stefan.jakovljevic@fmn.kg.ac.rs (S.J.); nenad.zornic@fmn.kg.ac.rs (N.Z.); stevan.eric@fmn.kg.ac.rs (S.E.); 2 Department of Vascular Surgery, University Clinical Center Kragujevac, 34000 Kragujevac, Serbia; 3 Department of Biochemistry, Faculty of Medical Sciences, University of Kragujevac, 34000 Kragujevac, Serbia; marina.mitrovic@fmn.kg.ac.rs (M.M.); petar.canovic@fmn.kg.ac.rs (P.Č.); marija.andjelkovic@fmn.kg.ac.rs (M.A.); 4 Department of Gynecology and Obstetrics, General Hospital Užice, 31000 Užice, Serbia; jevticmirela@fmn.kg.ac.rs; 5 Faculty of Medical Sciences, University of Kragujevac, 34000 Kragujevac, Serbia; 6 Clinic for Pulmonology, University Clinical Center Kragujevac, 34000 Kragujevac, Serbia; 7 Department of General Surgery, University Clinical Center Kragujevac, 34000 Kragujevac, Serbia; 8 Department of Pharmacology and Toxicology, Faculty of Medical Sciences, University of Kragujevac, 34000 Kragujevac, Serbia; marrina2006kg@yahoo.com; 9 Center for Research on Harmful Effects of Biological and Chemical Hazards, Faculty of Medical Sciences, University of Kragujevac, 34000 Kragujevac, Serbia; 10 Department of Internal Medicine, Faculty of Medical Sciences, University of Kragujevac, 34000 Kragujevac, Serbia; jelena.zivic@fmn.kg.ac.rs (J.Ž.); jelena.nesic@fmn.kg.ac.rs (J.N.); 11 Clinic for Gastroenterology and Hepatology, University Clinical Center Kragujevac, 34000 Kragujevac, Serbia; 12 Department of Endocrinology, University Clinical Center Kragujevac, 34000 Kragujevac, Serbia; 13 Department of Anesthesiology and Reanimation, University Clinical Center Kragujevac, 34000 Kragujevac, Serbia; 14 Department of Science, Institute for Information Technologies, University of Kragujevac, 34000 Kragujevac, Serbia; jmuskinja@kg.ac.rs; 15 Department of Gynecology and Obstetrics, Faculty of Medical Sciences, University of Kragujevac, 34000 Kragujevac, Serbia; sorakmarijakg@gmail.com; 16 Department of Biomedically Assisted Fertilization, Clinic of Gynecology and Obstetrics, University Clinical Center Kragujevac, 34000 Kragujevac, Serbia

**Keywords:** enone salts, mitochondrial apoptosis, HCT-116, HeLa, vanillin derivatives

## Abstract

Recent advances in enone chemistry have enabled the development of structurally optimized derivatives with improved anticancer selectivity. In this study, the cytotoxic activity and underlying mechanisms of sodium salts of four α,β-unsaturated enones (ES1–ES4), synthesized from vanillin-based scaffolds, were evaluated in human colorectal carcinoma (HCT-116), cervical adenocarcinoma (HeLa), and normal lung fibroblast (MRC-5) cell lines. All compounds exhibited concentration- and time-dependent cytotoxicity, with ES2 showing the highest potency (IC_50_ = 14.25 μM in HCT-116 and 18.12 μM in HeLa at 72 h) and minimal toxicity toward MRC-5 cells (IC_50_ > 90 μM). Although cisplatin demonstrated greater overall cytotoxicity, the enone salts displayed significantly higher selectivity indices, indicating a more favorable therapeutic window. Phase-contrast microscopy revealed characteristic morphological features of apoptosis, including cell rounding and membrane blebbing. Mechanistic investigations confirmed mitochondrial-mediated apoptosis, evidenced by increased early and late apoptotic populations, Bax upregulation, Bcl-2 downregulation, and caspase-3 activation. JC-10 staining demonstrated mitochondrial membrane depolarization accompanied by cytochrome c release. In addition, cell cycle analysis revealed pronounced G2/M phase arrest, particularly in HCT-116 cells. Collectively, these findings indicate that vanillin-derived enone sodium salts exert selective anticancer effects through mitochondrial apoptosis and cell cycle disruption, supporting their potential as low-toxicity anticancer candidates.

## 1. Introduction

The ongoing search for anticancer agents endowed with greater specificity and reduced systemic toxicity remains a central objective in oncology drug discovery, increasingly fueling investigations into structurally optimized natural product derivatives [1,2]. Among these, α,β-unsaturated enones have emerged as privileged molecular scaffolds due to their inherent electrophilic character, which enables selective Michael addition to nucleophilic amino acid residues, such as cysteines, within regulatory proteins implicated in tumorigenic signaling cascades [3,4]. This reactivity underpins their established activity in modulating cancer-related pathways and has positioned enones as versatile leads in medicinal chemistry programs [3,5].

Vanillin-based derivatives and dehydrozingerone analogs, derived from naturally occurring phenolic compounds, have demonstrated diverse biological activities, including antiproliferative effects in specific cancer models. For instance, dehydrozingerone (DZG) was evaluated in castration-resistant prostate cancer (CRPC) using the PLS10 cell line, where it exhibited an IC_50_ value of 153.13 ± 11.79 µM, while curcumin showed significantly higher potency (IC_50_ = 20.33 ± 0.58 µM). Additionally, vanillin-based complexes have been tested against human hepatocarcinoma (HUH-7), murine melanoma (B16–F10), and human renal adenocarcinoma (786-0) cell lines, demonstrating enhanced cytotoxic activity compared to vanillin alone [6,7]. Comprehensive structure–activity relationship (SAR) and mechanistic studies consistently highlight that rational β-position substitution using alkyl or aryl moieties, which profoundly influences both the potency and selectivity profiles of enone frameworks [8,9]. For example, strategic modifications at this position can alter lipophilicity, cell permeability, and molecular target affinity, thereby fine-tuning oncologic efficacy [10].

Building on foundational reports, four recently synthesized vanillin-derived enones (E1–E4), each functionalized at the β-position with methyl, isopropyl, isobutyl, or cyclopropyl groups, have shown compelling in vitro activity: these molecules induce apoptosis and G_2_/M cell cycle arrest in colon (HCT-116) and cervical (HeLa) cancer cells, with marked selectivity and minimal toxicity toward normal fibroblasts [11]. Despite this promise, hydrophobicity and poor aqueous solubility pose significant formulation and translational barriers, common to many small-molecule therapeutics [12,13,14].

Conversion of carboxylic acid-bearing pharmacophores to their sodium salt forms is a cornerstone of pharmaceutical science, proven to significantly enhance water solubility, improve oral and parenteral bioavailability, and facilitate favorable pharmacokinetic and pharmacodynamic profiles [15,16]. In addition, the ionic nature imparted by salt formation may augment compound–membrane interactions and cellular uptake, potentially expanding biological efficacy and permitting subsequent development into injectable or advanced delivery systems [17].

Motivated by these precedents, we synthesized the sodium salt derivatives ES1–ES4. We comprehensively evaluated their in vitro anticancer activity alongside mechanistic endpoints, which include effects on apoptosis pathways (Bax/Bcl-2 modulation, mitochondrial depolarization, caspase-3 activation) and cell cycle dynamics. To our knowledge, this is the first study to provide an integrated mechanistic assessment of sodium enone salts derived from vanillin within cancer cellular models. We hypothesized that conversion to the sodium salt form would at least maintain, if not enhance, antitumor efficacy and preserve selectivity for malignant over healthy cells, thus supporting the translational development of these optimized enone analogs as potential therapeutic candidate.

## 2. Results

### 2.1. Cytotoxic Effects of Enone Sodium Salts (ES1–ES4)

**Figure 1:**
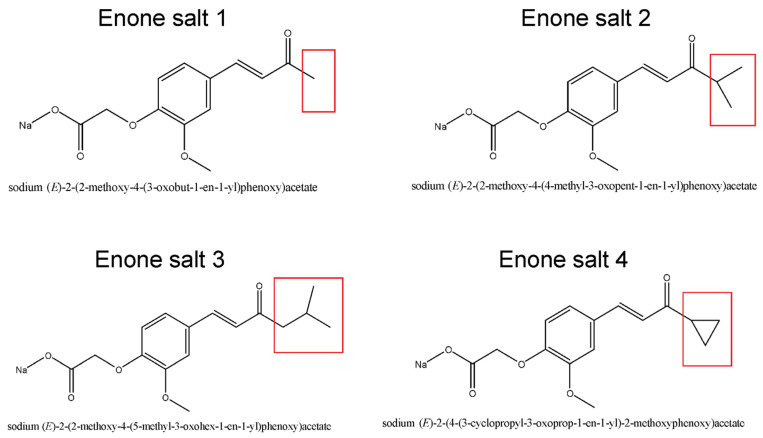
Chemical structures of sodium salts of α,β-unsaturated enones (ES1–ES4). ES1–enone salt 1 (methyl substituent); ES2–enone salt 2 (isopropyl substituent); ES3–enone salt 3 (isobutyl substituent); ES4–enone salt 4 (cyclopropyl substituent). The red boxes highlight the variable substituents of the enone moiety.

The chemical structures of the sodium salts of α,β-unsaturated enones (ES1–ES4) are presented in Figure 1.

**Figure 2:**
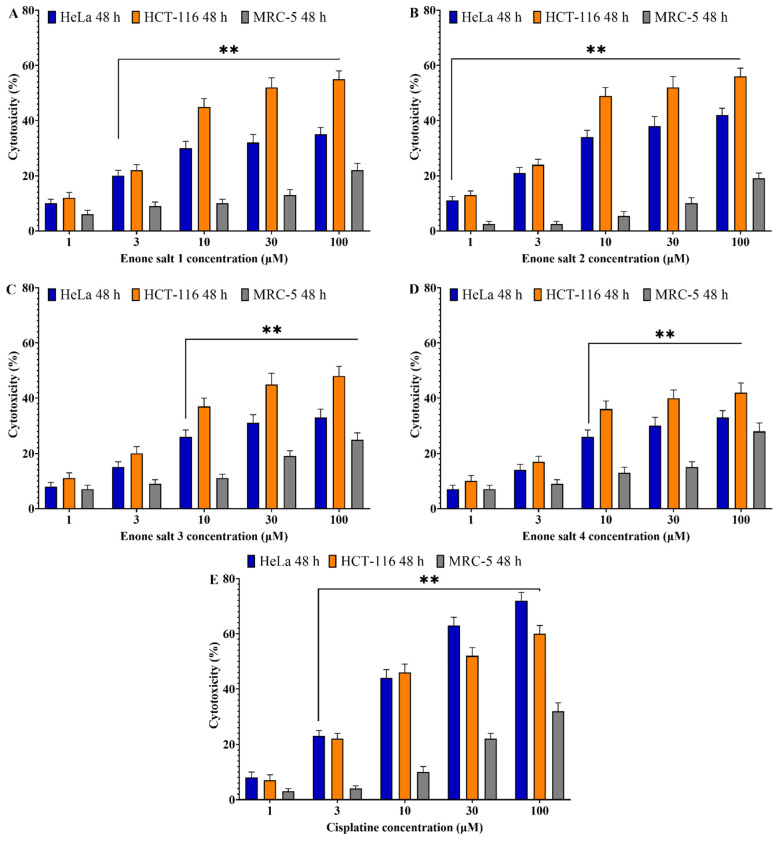
Cytotoxic effects of enone sodium salts (ES1–ES4) and cisplatin on HCT-116 (colon cancer), HeLa (cervical cancer), and MRC-5 (normal lung fibroblast) cells after 48 h of treatment. (**A**) Enone salt 1 (ES1); (**B**) Enone salt 2 (ES2); (**C**) Enone salt 3 (ES3); (**D**) Enone salt 4 (ES4); (**E**) Cisplatin. Cell viability was determined using the MTT assay. Data are presented as mean ± SD of three independent experiments. Cytotoxicity (%) was calculated as: Cytotoxicity (%) = [1 − (Absorbance of treated cells/Absorbance of control cells)] × 100. ** indicates statistical significance at *p* < 0.01 compared to untreated control cells.

**Figure 3:**
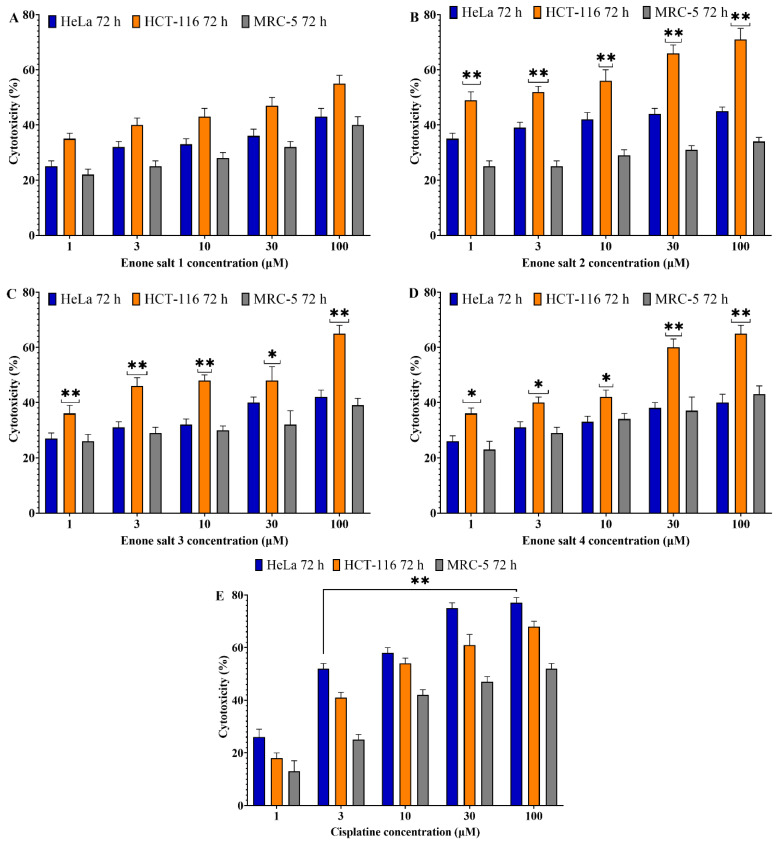
Cytotoxic effects of enone sodium salts (ES1–ES4) and cisplatin on HCT-116 (colon cancer), HeLa (cervical cancer), and MRC-5 (normal lung fibroblast) cells after 72 h of treatment. (**A**) Enone salt 1 (ES1); (**B**) Enone salt 2 (ES2); (**C**) Enone salt 3 (ES3); (**D**) Enone salt 4 (ES4); (**E**) Cisplatin. Cell viability was determined using the MTT assay. Data are presented as mean ± SD of three independent experiments. Cytotoxicity (%) was calculated as: Cytotoxicity (%) = [1 − (Absorbance of treated cells/Absorbance of control cells)] × 100. * indicates statistical significance at *p* < 0.05 compared to untreated control cells. ** indicates statistical significance at *p* < 0.01 compared to untreated control cells.

To evaluate the antiproliferative properties of enone sodium salts, MTT assays were conducted on HCT-116 (colon adenocarcinoma), HeLa (cervical carcinoma), and MRC-5 (normal lung fibroblast) cell lines following treatment with ES1–ES4 at concentrations of 1, 3, 10, 30, and 100 μM. Cytotoxic effects were assessed after 48 and 72 h of incubation. All tested compounds induced significant, concentration-dependent cytotoxicity in HCT-116 and HeLa cells at both time points, while exerting minimal toxicity toward MRC-5 cells (Figure 2 and Figure 3).

The cytotoxic response was more pronounced after 72 h, with HCT-116 cells showing the highest sensitivity to treatment. At the maximum concentration tested (100 μM), all enone salts reduced HCT-116 cell viability by more than 70%. In contrast, MRC-5 cells exhibited substantially lower sensitivity under the same conditions, with viability typically remaining above 50%.

Among the tested compounds, ES2 demonstrated the most potent cytotoxic effect across both cancer cell lines, followed by ES1. Cytotoxic effects of ES3 and ES4 were slightly less pronounced but still statistically significant.

These findings indicate that ES1–ES4 possess substantial and time-dependent cytotoxic activity against malignant cells while maintaining low toxicity toward normal fibroblasts.

### 2.2. IC_50_ Values of Enone Salts

The half-maximal inhibitory concentrations (IC_50_) of ES1–ES4 and cisplatin were determined after 48 and 72 h of treatment in HCT-116, HeLa, and MRC-5 cells (Table 1). All enone sodium salts exhibited lower IC_50_ values in cancer cell lines compared to normal fibroblasts, yielding Selectivity Index (SI) values at 72 h ranging from approximately 5.1 to 6.4 in HCT-116 cells, confirming a favorable degree of tumor selectivity.

IC_50_ values (µM) were determined from MTT assay data for HCT-116 (colon carcinoma), HeLa (cervical carcinoma), and MRC-5 (normal lung fibroblast) cells following 48 h and 72 h exposure to enone sodium salts (ES1–ES4) and cisplatin (CisPt). Data are presented as mean ± SD of three independent experiments. The Selectivity Index (SI) was calculated for 72 h exposure as: SI = IC_50_ (MRC-5)/IC_50_ (cancer cells).

At 48 h, the most potent compound against HCT-116 cells was ES2 (30.94 ± 2.11 μM), followed closely by ES1 (32.66 ± 1.82 μM). In HeLa cells, IC_50_ values for ES1 and ES2 were 51.34 ± 0.19 μM and 49.76 ± 1.22 μM, respectively. Notably, the IC_50_ values in MRC-5 cells for all enone salts exceeded 90 μM, indicating reduced cytotoxicity toward normal cells.

After 72 h, a time-dependent enhancement of cytotoxicity was observed. IC_50_ values in HCT-116 cells dropped to 14.25–15.65 μM for all tested salts, with ES2 showing the lowest value (14.25 ± 0.78 μM). A similar trend was noted in HeLa cells, where IC_50_ values ranged from 18.12 ± 0.93 μM (ES2) to 29.05 ± 1.43 μM (ES4). In contrast, MRC-5 IC_50_ values remained above 75 μM at all time points.

The selectivity index (SI), calculated as the ratio of IC_50_ in MRC-5 to IC_50_ in tumor cells, further supported the therapeutic potential of these compounds. The calculated Selectivity Index (SI) values at 72 h ranged from 5.1 to 6.4 for HCT-116 cells and from 2.9 to 5.0 for HeLa cells (Table 1), indicating a favorable degree of tumor selectivity. These findings emphasize the time-dependent potency and selective cytotoxicity of ES1–ES4 toward malignant cells.

### 2.3. Morphological Changes in Treated Cancer Cells

**Figure 4:**
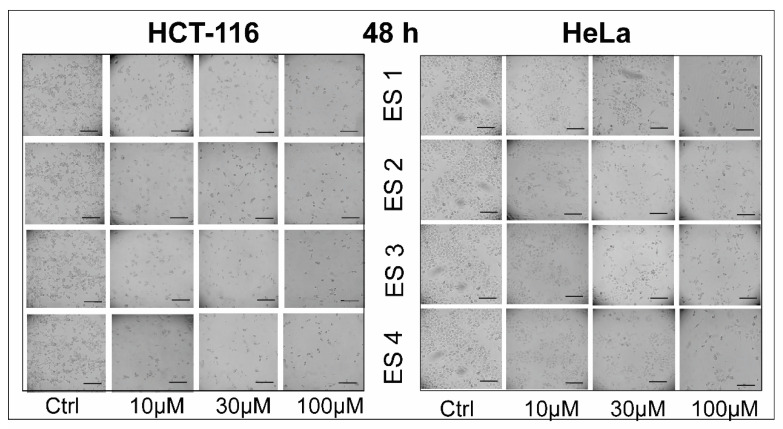
Morphological changes in HCT-116 and HeLa cells following 48 h of treatment with enone sodium salts (ES1–ES4) at various concentrations (10, 30, and 100 μM). Cellular morphology was assessed using phase-contrast microscopy. Treated cells exhibited features indicative of cytotoxic damage, including membrane blebbing, rounding, and detachment, in a dose-dependent manner. Scale bar = 100 μm.

**Figure 5:**
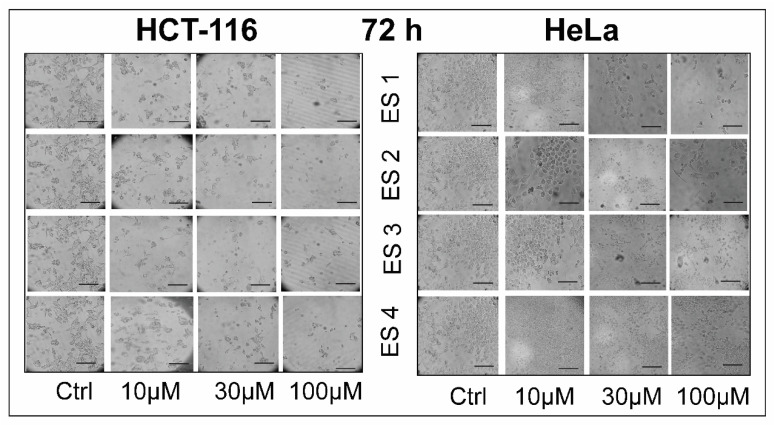
Morphological changes in HCT-116 and HeLa cells following 72 h of treatment with enone sodium salts (ES1–ES4) at various concentrations (10, 30, and 100 μM). Cellular morphology was assessed using phase-contrast microscopy. Treated cells exhibited features indicative of cytotoxic damage, including membrane blebbing, rounding, and detachment, in a dose-dependent manner. Scale bar = 100 μm.

Morphological assessment of HCT-116 and HeLa cells was performed following 48 and 72 h of treatment with enone sodium salts (ES1–ES4) at concentrations of 10, 30, and 100 μM. Phase-contrast microscopy revealed clear dose- and time-dependent alterations in cellular architecture (Figure 4 and Figure 5).

After 48 h of exposure, cells displayed early morphological signs of cytotoxic damage, including rounding, membrane blebbing, and partial detachment from the culture surface. These effects were most prominent at 100 μM for ES1 and ES2. At lower concentrations, cells exhibited subtle changes in shape and contact inhibition. Following 72 h of treatment, these alterations became more pronounced. A marked reduction in cell density, severe membrane disintegration, and loss of intercellular adherence were observed, particularly in ES2- and ES1-treated samples. In contrast, control cells retained normal spindle-like morphology with intact monolayers.

These findings suggest that ES1–ES4 exert progressive cytotoxic effects on cancer cells over time, consistent with viability data from the MTT assay.

### 2.4. Induction of Apoptosis by Enone Salts

**Figure 6:**
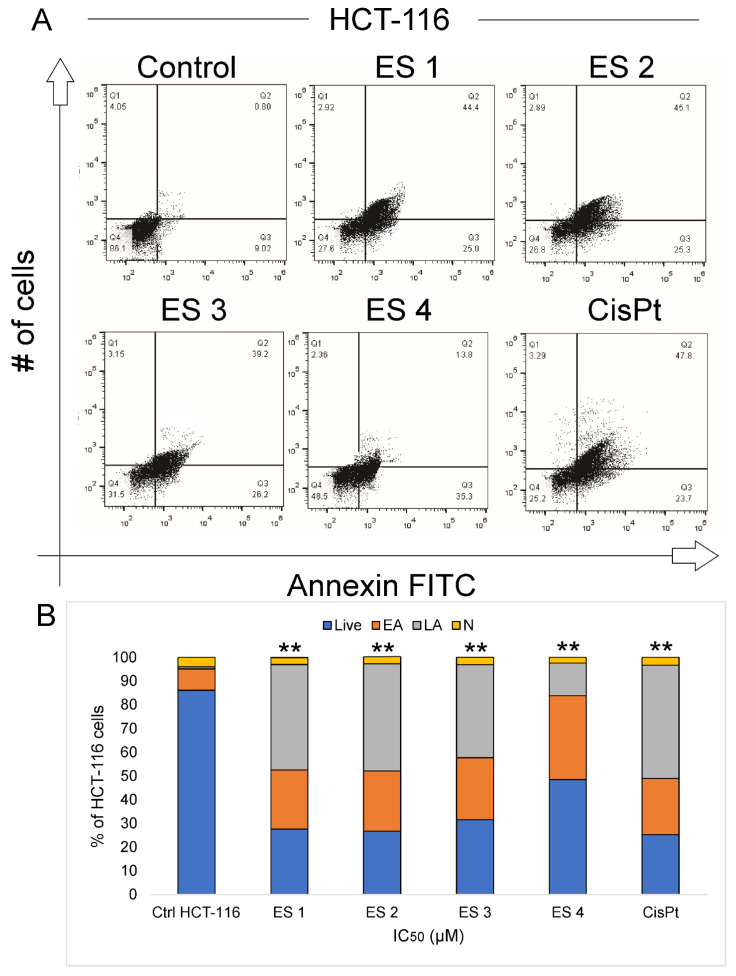
Induction of apoptosis in HCT-116 cells following 48 h treatment with enone sodium salts (ES1–ES4) at their IC_50_ concentrations, assessed by Annexin V-FITC/7-AAD staining and flow cytometry. (**A**) Representative dot plots showing viable (Annexin V^−^/7-AAD^−^), early apoptotic (Annexin V^+^/7-AAD^−^), late apoptotic (Annexin V^+^/7-AAD^+^), and necrotic (Annexin V^−^/7-AAD^+^) cell populations in control and treated cells. (**B**) Quantitative analysis of cell populations expressed as percentages of total HCT-116 cells. Live—viable cells; EA—early apoptosis; LA—late apoptosis; N—necrotic cells. Data are presented as mean ± SD of three independent experiments. ** indicates statistical significance at *p* < 0.01 vs. untreated control (ANOVA followed by Tukey’s post hoc test).

**Figure 7:**
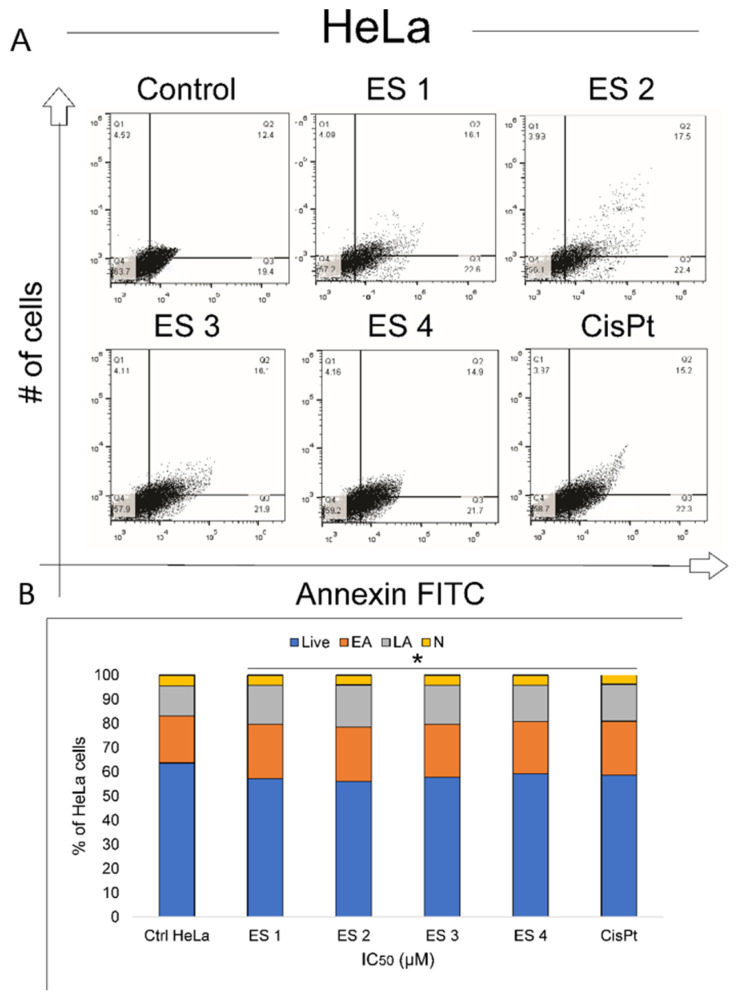
Induction of apoptosis in HeLa cells following 48 h treatment with enone sodium salts (ES1–ES4) at their IC_50_ concentrations, assessed by Annexin V-FITC/7-AAD staining and flow cytometry. (**A**) Representative dot plots showing viable (Annexin V^−^/7-AAD^−^), early apoptotic (Annexin V^+^/7-AAD^−^), late apoptotic (Annexin V^+^/7-AAD^+^), and necrotic (Annexin V^−^/7-AAD^+^) cell populations in control and treated cells. (**B**) Quantitative analysis of cell populations expressed as percentages of total HeLa cells. Live—viable cells; EA—early apoptosis; LA—late apoptosis; N—necrotic cells. Data are presented as mean ± SD of three independent experiments. * *p* < 0.05 vs. untreated control (ANOVA followed by Tukey’s post hoc test).

To determine whether the cytotoxic effects of ES1–ES4 were associated with apoptosis, Annexin V-FITC/7-AAD dual staining was performed on HCT-116 and HeLa cells after 48 h of treatment at their respective IC_50_ concentrations. Flow cytometry analysis revealed significant increases in both early and late apoptotic cell populations in treated samples compared to controls (Figure 6 and Figure 7).

In HCT-116 cells, treatment with ES2 induced the highest percentage of apoptotic cells, followed by ES1, ES3, and ES4. The majority of apoptotic cells were in the early phase, consistent with activation of the apoptotic program. HeLa cells displayed a similar trend, though the proportion of apoptotic cells was generally lower compared to HCT-116.

All enone salts induced statistically significant increases in total apoptosis in both cell lines. The apoptotic response was strongest in ES2-treated cells, aligning with their potent cytotoxic and morphological effects observed in previous assays.

These findings suggest that apoptosis is a key mechanism of cell death induced by ES1–ES4 in both colon and cervical cancer cells.

### 2.5. Modulation of Bax and Bcl-2 Expression

**Figure 8:**
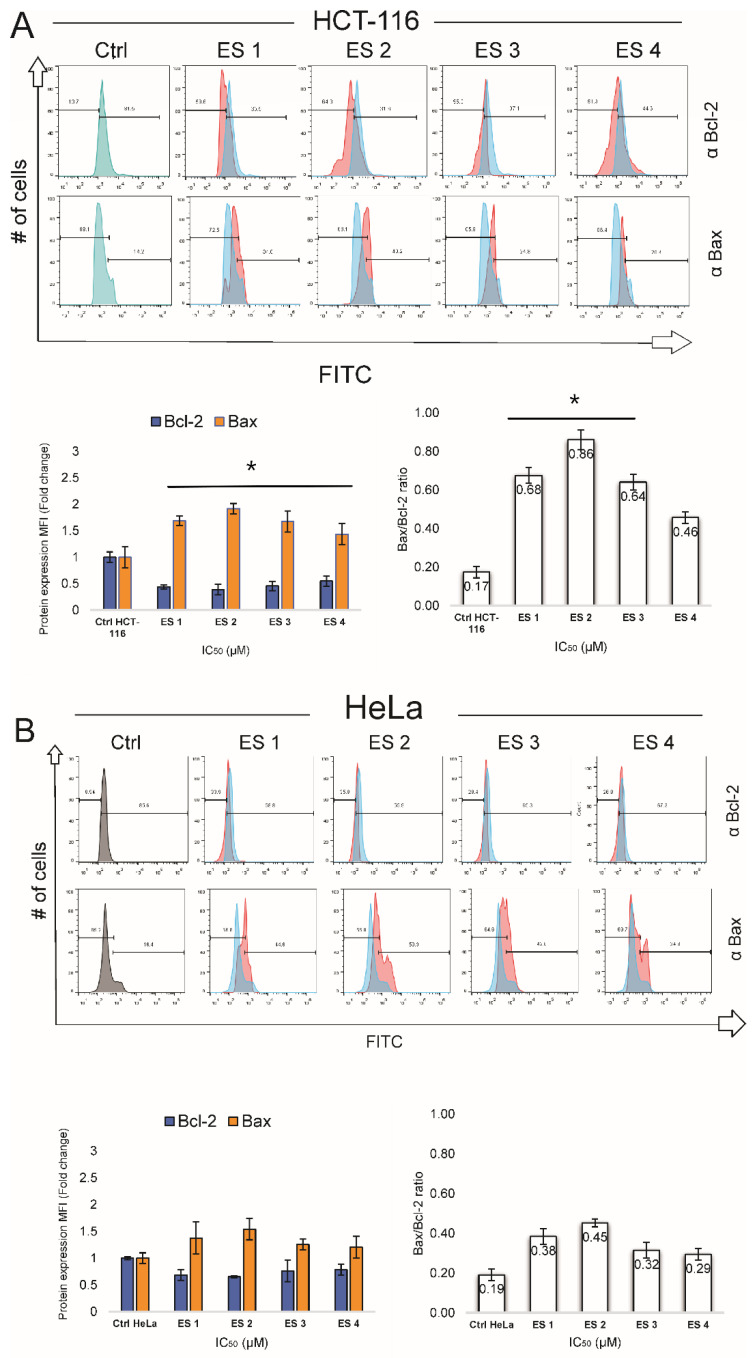
Expression of pro-apoptotic Bax and anti-apoptotic Bcl-2 proteins in HCT-116 and HeLa cells following 48 h treatment with enone sodium salts (ES1–ES4) at their IC_50_ concentrations. (**A**) Representative flow cytometry histograms showing intracellular expression of Bcl-2 (**upper row**) and Bax (**lower row**) in HCT-116 cells, where blue histograms represent untreated control cells and red histograms represent treated cells. Bar graphs present relative mean fluorescence intensity (MFI) values of Bcl-2 and Bax (**left graph**) and the calculated Bax/Bcl-2 ratio (**right graph**). (**B**) Representative flow cytometry histograms showing intracellular expression of Bcl-2 (**upper row**) and Bax (**lower row**) in HeLa cells, where blue histograms represent untreated control cells and red histograms represent treated cells. Bar graphs present relative mean fluorescence intensity (MFI) values of Bcl-2 and Bax (**left graph**) and the calculated Bax/Bcl-2 ratio (**right graph**). Data are presented as mean ± SD of three independent experiments. * *p* < 0.05 vs. untreated control (one-way ANOVA followed by Tukey’s post hoc test).

To further elucidate the mechanism of apoptosis induction, the expression of key regulatory proteins Bax and Bcl-2 was analyzed by flow cytometry after 48 h treatment with enone sodium salts (ES1–ES4) at their respective IC_50_ concentrations (Figure 8). In HCT-116 cells, all tested compounds significantly increased the expression of the pro-apoptotic protein Bax, with mean fluorescence intensity (MFI) values ranging from 1.4 to 1.9. Concurrently, a reduction in the anti-apoptotic protein Bcl-2 was observed, contributing to a shift in the apoptotic balance toward cell death. The most pronounced changes were induced by ES2, followed by ES1, ES3, and ES4.

A similar trend was observed in HeLa cells, though the magnitude of Bax upregulation and Bcl-2 suppression was less marked compared to HCT-116. ES1 and ES2 elicited the strongest pro-apoptotic protein expression patterns in this cell line.

These results indicate that ES1–ES4 modulate apoptotic signaling at the protein level, favoring mitochondrial-mediated apoptosis through increased Bax expression and suppression of Bcl-2.

### 2.6. Activation of Caspase-3

**Figure 9:**
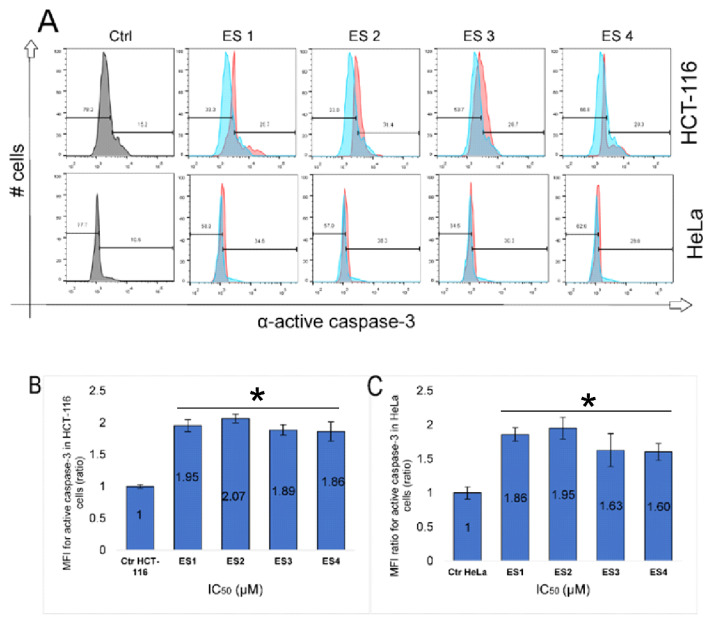
Expression of cleaved (active) caspase-3 in HCT-116 and HeLa cells following 48 h treatment with enone sodium salts (ES1–ES4) at their IC_50_ concentrations. (**A**) Representative flow cytometry histograms showing intracellular levels of active caspase-3 in HCT-116 (**upper panel**) and HeLa (**lower panel**) cells, where blue histograms represent untreated control cells and red histograms represent treated cells. A rightward shift of the red histogram indicates increased activation of caspase-3. (**B**) Quantitative analysis of mean fluorescence intensity (MFI) of active caspase-3 in HCT-116 cells, expressed as fold change relative to untreated control. (**C**) Quantitative analysis of mean fluorescence intensity (MFI) of active caspase-3 in HeLa cells, expressed as fold change relative to untreated control. Data are presented as mean ± SD of three independent experiments. * *p* < 0.05 vs. untreated control (one-way ANOVA followed by Tukey’s post hoc test).

To confirm the execution phase of apoptosis, the expression of cleaved (active) caspase-3 was evaluated in HCT-116 and HeLa cells following 48 h treatment with enone sodium salts (ES1–ES4) at their respective IC_50_ concentrations. Flow cytometric analysis revealed a significant increase in caspase-3 activation in both cell lines compared to untreated controls (Figure 9).

In HCT-116 cells, the most pronounced increase in cleaved caspase-3 expression was observed in ES2-treated cells, followed by ES1, ES3, and ES4. The fold-change in MFI ranged from 1.86 to 2.07. HeLa cells displayed a similar activation profile, with ES2 and ES1 again inducing the highest levels of caspase-3 activity (fold-change ~1.6 to 2.0), while ES3 and ES4 exhibited slightly lower effects.

These findings provide further evidence that ES1–ES4 trigger apoptosis through the intrinsic pathway and confirm the involvement of executioner caspase-3 as a downstream effector of cell death in both colon and cervical cancer cells.

### 2.7. Disruption of Mitochondrial Membrane Potential (ΔΨm)

To determine whether enone sodium salts induce mitochondrial dysfunction, changes in mitochondrial membrane potential (ΔΨm) were assessed using the JC-10 fluorescent dye in HCT-116 and HeLa cells after 48 h treatment at IC_50_ concentrations. In healthy cells, JC-10 forms red fluorescent aggregates within intact mitochondria, whereas mitochondrial depolarization results in green-fluorescent monomers in the cytosol.

**Figure 10:**
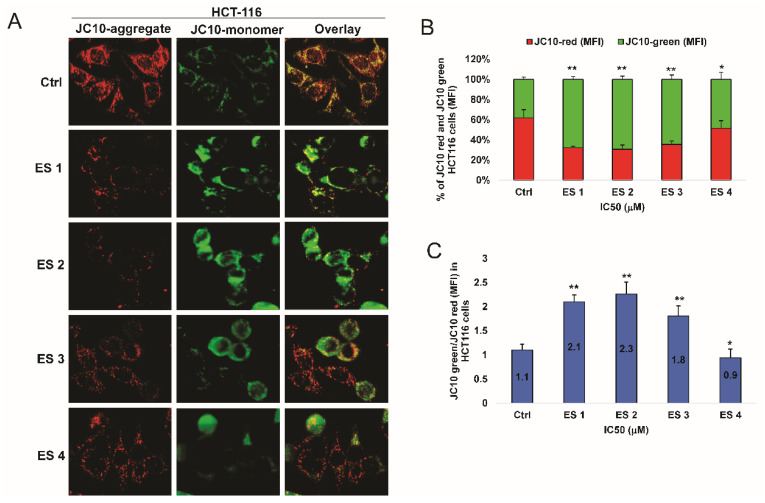
Disruption of mitochondrial membrane potential (ΔΨm) in HCT-116 cells following 48 h treatment with enone sodium salts (ES1–ES4) at their IC_50_ concentrations, assessed using the JC-10 fluorescent probe. (**A**) Representative fluorescence images showing JC-10 aggregates (red fluorescence) in polarized mitochondria and JC-10 monomers (green fluorescence) in depolarized mitochondria. Overlay images illustrate the shift from red to green fluorescence following treatment. (**B**) Quantitative analysis of JC-10 red and JC-10 green mean fluorescence intensity (MFI) expressed as percentage of total fluorescence signal. (**C**) Ratio of JC-10 green/red MFI in HCT-116 cells, reflecting mitochondrial depolarization. Data are presented as mean ± SD of three independent experiments. * *p* < 0.05, ** *p* < 0.01 vs. untreated control (ANOVA followed by Tukey’s post hoc test).

**Figure 11:**
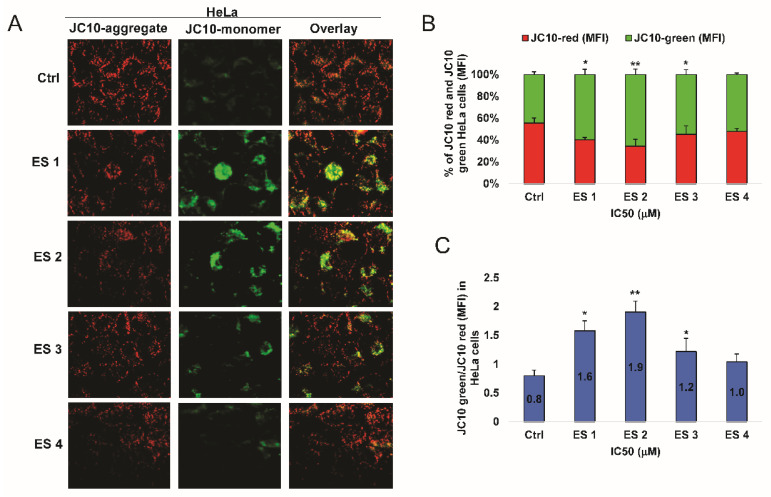
Disruption of mitochondrial membrane potential (ΔΨm) in HeLa cells following 48 h treatment with enone sodium salts (ES1–ES4) at their IC_50_ concentrations, assessed using the JC-10 fluorescent probe. (**A**) Representative fluorescence images showing JC-10 aggregates (red fluorescence) in polarized mitochondria and JC-10 monomers (green fluorescence) in depolarized mitochondria. Overlay images illustrate the shift from red to green fluorescence following treatment. (**B**) Quantitative analysis of JC-10 red and JC-10 green mean fluorescence intensity (MFI) expressed as percentage of total fluorescence signal in HeLa cells. (**C**) Ratio of JC-10 green/red MFI in HeLa cells, reflecting mitochondrial depolarization. Data are presented as mean ± SD of three independent experiments. * *p* < 0.05, ** *p* < 0.01 vs. untreated control (ANOVA followed by Tukey’s post hoc test).

Fluorescence microscopy revealed a notable shift from red to green fluorescence in both cancer cell lines following treatment with ES1–ES4 (Figure 10 and Figure 11). This fluorescence transition was quantified by calculating the ratio of green to red signal intensity.

In HCT-116 cells, ES2 induced the most pronounced depolarization, with a JC-10 green/red ratio 2.3, followed by ES1, ES3, and ES4.

Similar results were observed in HeLa cells, although the overall magnitude of ΔΨm disruption was slightly lower, with green/red ratios ranging from 1.0 to 1.9.

These data indicate that treatment with ES1–ES4 leads to a loss of mitochondrial membrane potential in both cancer cell types, supporting the involvement of the intrinsic apoptotic pathway in their mechanism of action.

### 2.8. Translocation of Cytochrome c

To further validate the involvement of mitochondrial-mediated apoptosis, the intracellular localization of cytochrome c was assessed by immunofluorescence following 48 h treatment of HCT-116 and HeLa cells with enone sodium salts (ES1–ES4) at IC_50_ concentrations.

**Figure 12:**
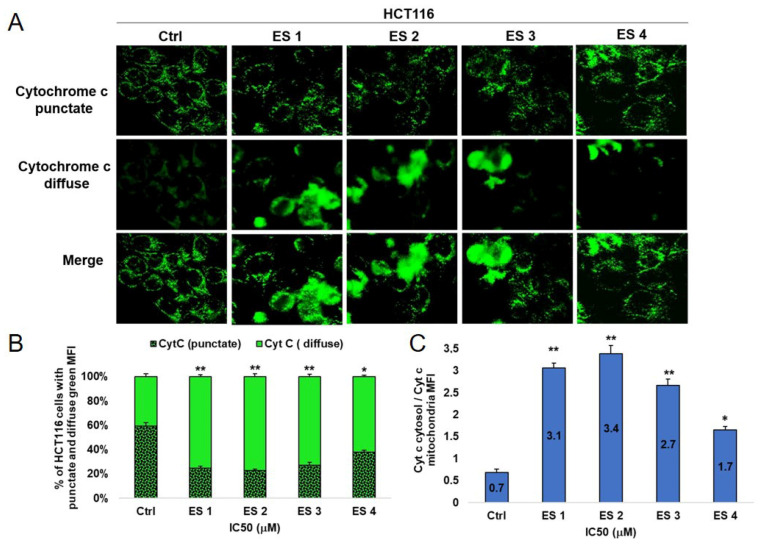
Expression and subcellular localization of cytochrome c in HCT-116 cells following 48 h treatment with enone sodium salts (ES1–ES4) at their IC_50_ concentrations. (**A**) Representative immunofluorescence images showing redistribution of cytochrome c from mitochondria (punctate pattern) to cytoplasm (diffuse staining) in treated cells, indicating mitochondrial membrane permeabilization. Scale bar = 20 μm. (**B**) Quantitative analysis of HCT-116 cells exhibiting punctate and diffuse green fluorescence patterns, expressed as percentages of total analyzed cells. (**C**) Ratio of cytosolic to mitochondrial cytochrome c mean fluorescence intensity (MFI). Data are presented as mean ± SD of three independent experiments. * *p* < 0.05, ** *p* < 0.01 vs. untreated control (ANOVA followed by Tukey’s post hoc test).

**Figure 13:**
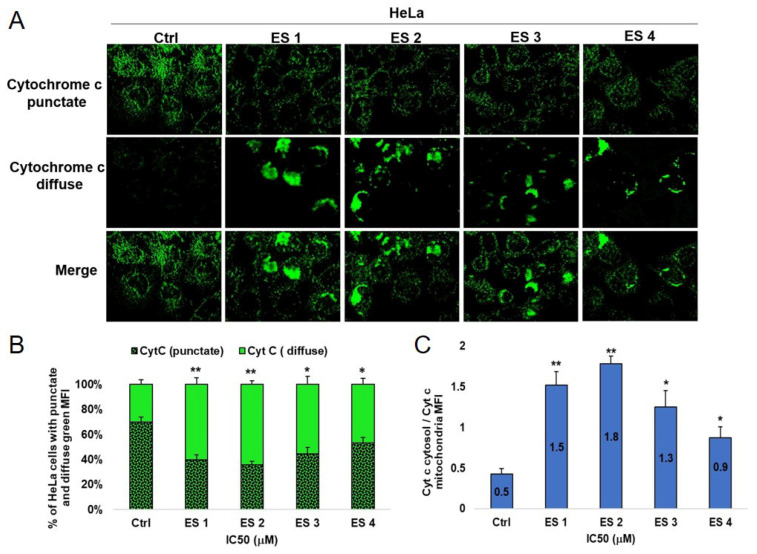
Expression and subcellular localization of cytochrome c in HeLa cells following 48 h treatment with enone sodium salts (ES1–ES4) at their IC_50_ concentrations. (**A**) Representative immunofluorescence images showing redistribution of cytochrome c from mitochondria (punctate pattern) to cytoplasm (diffuse staining) in treated cells, indicating mitochondrial membrane permeabilization. Scale bar = 20 μm. (**B**) Quantitative analysis of HeLa cells exhibiting punctate and diffuse green fluorescence patterns, expressed as percentages of total analyzed cells. (**C**) Ratio of cytosolic to mitochondrial cytochrome c mean fluorescence intensity (MFI). Data are presented as mean ± SD of three independent experiments. * *p* < 0.05, ** *p* < 0.01 vs. untreated control (ANOVA followed by Tukey’s post hoc test).

In untreated control cells, cytochrome c staining appeared punctate and perinuclear, consistent with mitochondrial localization. In contrast, ES1–ES4 treatment led to a marked redistribution of cytochrome c into the cytoplasm, as indicated by diffuse green fluorescence (Figure 12 and Figure 13).

The extent of cytochrome c release varied between compounds. In HCT-116 cells, ES2 caused the most pronounced cytoplasmic dispersion, followed by ES1, ES3, and ES4.

HeLa cells exhibited similar translocation patterns, although the intensity of cytosolic cytochrome c was lower compared to HCT-116.

These findings suggest that ES1–ES4 trigger mitochondrial outer membrane permeabilization and cytochrome c release, reinforcing the activation of the intrinsic apoptotic cascade in both cancer cell lines.

### 2.9. Cell Cycle Arrest Induced by Enone Salts

**Figure 14:**
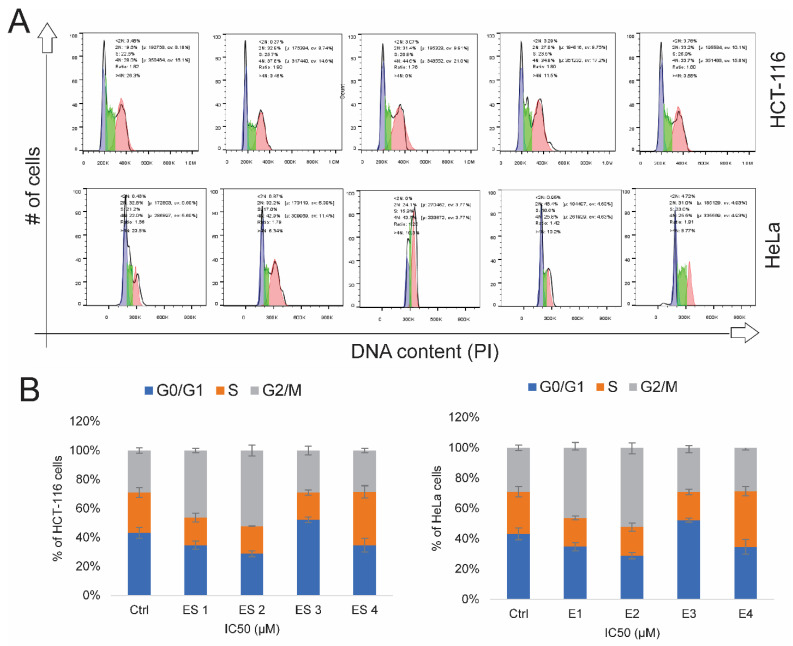
Cell cycle distribution of HCT-116 and HeLa cells following 48 h treatment with enone sodium salts (ES1–ES4) at their IC_50_ concentrations, assessed by propidium iodide (PI) staining and flow cytometry. (**A**) Representative DNA content histograms showing cell cycle phase distribution (G0/G1, S, and G2/M) in HCT-116 (**upper panel**) and HeLa (**lower panel**) cells. (**B**) Quantitative analysis of the percentage of cells in G0/G1, S, and G2/M phases in HCT-116 (**left graph**) and HeLa (**right graph**) cells following treatment. Data are presented as mean ± SD of three independent experiments. Statistical significance was determined by one-way ANOVA followed by Tukey’s post hoc test.

To investigate the effect of enone sodium salts on cell cycle progression, HCT-116 and HeLa cells were treated with IC_50_ concentrations of ES1–ES4 for 48 h and analyzed by flow cytometry. DNA content was measured to determine the distribution of cells across G_0_/G_1_, S, and G_2_/M phases (Figure 14).

In HCT-116 cells, all compounds induced a clear accumulation in the G_2_/M phase. ES2 elicited the most pronounced effect, increasing the G_2_/M fraction from 28% in control cells to 44.6%. ES1, ES3, and ES4 similarly elevated the G_2_/M population to 37.8%, 34.8%, and 33.7%, respectively. These changes were accompanied by a modest increase in the G_0_/G_1_ population, suggesting a delay in cell cycle progression.

In HeLa cells, a comparable trend was observed. ES2 and ES1 produced the most substantial G_2_/M arrest, increasing this fraction from 22% in control cells to 43.7% and 42.9%, respectively. ES3 and ES4 induced moderate increases in the G_2_/M phase (25.8% and 25.5%). Additionally, ES3 led to a marked elevation in G_0_/G_1_ phase (46.4%), indicating potential arrest at an earlier checkpoint. ES4 increased both G_0_/G_1_ (31%) and S phase (33%), suggesting a compound-specific impact on multiple phases.

These results confirm that enone salts interfere with normal cell cycle dynamics in both tumor cell lines, with a predominant arrest at the G_2_/M phase, consistent with antiproliferative activity.

## 3. Discussion

Cancer continues to pose a substantial threat to global health, with colorectal and cervical malignancies maintaining persistently high incidence and mortality rates across geographical regions [18]. While significant strides have been made in pharmacotherapy, therapeutic success is frequently undermined by drug resistance, off-target cytotoxicity, and poor pharmacokinetic profiles [19,20,21]. This persistent clinical challenge stimulates interest in discovering and optimizing chemical scaffolds that maximize antitumor efficacy while minimizing harm to healthy tissue.

In this context, the present study builds on and expands beyond the existing body of work on α,β-unsaturated enones derived from vanillin [22,23]. Vanillin-based enones are particularly attractive for anticancer research because they are easily derivatized and display a range of pharmacological properties, including antiproliferative, anti-inflammatory, and antioxidant activities [24]. To address issues of solubility which is a well-known limitation in the clinical progression of bioactive compounds [25], the present research developed sodium carboxylate salts (ES1–ES4) from parent enones (E1–E4), each characterized by a specific alkyl substituent (methyl, isopropyl, isobutyl, cyclopropyl). Salt formation represents a well-established pharmaceutical strategy aimed at improving aqueous solubility through ionization of the carboxyl functional group. Beyond enhancing dissolution behavior and facilitating formulation development, accumulating evidence suggests that salt conversion may also influence cellular uptake and, consequently, biological activity [25,26]. In the present study, however, quantitative solubility assessment was not formally performed and therefore remains to be addressed in future investigations.

### 3.1. Comparative Cytotoxicity of Sodium Salts and Broader Context

In the present study, ES1–ES4 displayed time-dependent cytotoxicity, with IC_50_ values at 48 h ranging from 30.94–77.11 µM in HCT-116 and 49.76–55.19 µM in HeLa cells. In our previous study [11], the parent enones (E1–E4) exhibited IC_50_ values ranging from 14.2–22.8 µM in HCT-116 cells and 17.9–25.6 µM in HeLa cells after 48 h of treatment. In contrast, the corresponding sodium salts evaluated in the present study showed IC_50_ values between 30.9–77.1 µM under comparable experimental conditions. These findings indicate that salt formation does not enhance intrinsic cytotoxic potency at 48 h, but rather preserves selective antitumor activity while potentially offering improved physicochemical and formulation properties. After 72 h, potency increased substantially, with IC_50_ values clustering around 14.25–15.65 µM in HCT-116 and 18.12–29.05 µM in HeLa (Table 1). Importantly, IC_50_ values in MRC-5 cells remained high (≥76 µM at 72 h and >90–>100 µM at 48 h), supporting a selectivity window toward malignant cells. Compared with the previously reported parent enones (E1–E4) evaluated under analogous experimental settings [11], the sodium carboxylates do not show improved potency at 48 h; however, they maintain a favorable selectivity profile toward cancer cells over MRC-5 fibroblasts, enable mechanistic interrogation in their salt form and can be contextualized within the broader landscape of contemporary alkylaminated and salt-based anticancer agents, including selenite salts and other sodium-based systems reported to exert anticancer effects through diverse mechanisms [27,28]. These findings reinforce the established principle that strategic salt formation is not merely a means of overcoming formulation challenges, but may also influence physicochemical behavior and downstream biological responses: it can directly influence biological outcomes by modulating dissolution rate, cellular uptake, and bioavailability [25,26].

To directly contextualize these findings, it is important to compare the present IC_50_ values with those previously reported for the corresponding parent enones (E1–E4) under comparable experimental conditions [11]. In the earlier study, the parent compounds demonstrated IC_50_ values in HCT-116 and HeLa cells that were generally lower than those observed here at the 48 h time point, indicating higher intrinsic cytotoxic potency. In contrast, the sodium salts (ES1–ES4) displayed slightly higher IC_50_ values at 48 h, suggesting that salt formation did not enhance early cytotoxic activity. However, after 72 h of exposure, the potency gap narrowed, with IC_50_ values for ES derivatives clustering around 14–16 μM in HCT-116 cells and 18–29 μM in HeLa cells. Importantly, the selectivity index remained favorable and comparable to that reported for the parent enones, as cytotoxicity toward MRC-5 fibroblasts remained substantially lower. These findings indicate that conversion to the sodium salt form preserves selective antitumor activity while primarily offering advantages related to physicochemical properties and potential formulation development rather than increased intrinsic cytotoxic potency.

When compared to cisplatin, the reference chemotherapeutic agent, the enone sodium salts exhibited lower intrinsic cytotoxic potency, particularly at 72 h, where cisplatin demonstrated IC_50_ values approximately two-fold lower in HCT-116 cells. However, the sodium salts maintained a favorable selectivity profile toward malignant cells, suggesting that their therapeutic potential may lie in optimized safety and formulation advantages rather than superior potency.

Cross-referencing other sodium-based or salt-form agents such as sodium selenite in cervical, pancreatic, and breast cancers, demonstrates that the shift to ionic forms enhances proapoptotic efficacy and may increase tumor selectivity [27]. Further, the concept of targeting cellular ionic balance is gaining attraction, with recent work demonstrating that boosting intracellular sodium via nanoparticles or salt complexes selectively kills tumor cells while sparing healthy tissue [29,30]. In the present study, selectivity was evident: IC_50_ values in healthy MRC-5 fibroblasts remained substantially higher than those observed in cancer cells (76.42–91.31 µM at 72 h and >90–>100 µM at 48 h), supporting a degree of selectivity toward malignant cells., suggesting a degree of selectivity consistent with criteria commonly applied in early-stage anticancer lead evaluation.

### 3.2. Roles and Optimization of Alkyl Substituents

Beyond the primary effects of salt formation, this work substantiates the critical impact of alkyl side chains in drug performance, echoing and extending findings from computational and medicinal chemistry studies [31,32,33]. Substituent size, branching, and electronic effects regulate a compound’s hydrophobicity, membrane permeability, binding affinity, and, ultimately, cytotoxicity [34,35,36]. The pronounced cytotoxicity of ES1 (methyl) and ES2 (isopropyl) among the tested analogs mirrors reports that branched or short-chain alkylations potentiate membrane passage and molecular interactions with cellular targets [31]. This SAR (structure–activity relationship) principle is not unique to vanillin enones; comparable trends are reported for chalcone analogs, alkylaminated icariside derivatives, and heteroaromatic cancer therapies [37]. Collectively, these observations validate the idea that fine-tuned substituent optimization remains a cornerstone of anticancer drug development.

### 3.3. Mechanistic Insights: Intrinsic Apoptosis, Mitochondrial Pathways, and Ionic Modulation

Mechanistic studies in this investigation reveal that ES1–ES4 primarily induce mitochondrial-driven apoptosis which is a mode of cell death prominent in the action of many contemporary anticancer drugs [38,39,40,41]. Flow cytometric quantification of annexin V/PI staining confirmed significant apoptosis (*p* < 0.01), accompanied by altered Bax/Bcl-2 ratios and marked loss of mitochondrial membrane potential (ΔΨm). Release of cytochrome c and activation of executioner caspase-3 signal progression through the classic intrinsic apoptosis cascade [42,43,44]. Notably, mitochondrial targeted therapies and nanotechnological approaches, which aim to disrupt energy metabolism and induce lethal oxidative stress, are gaining clinical momentum [45].

Parallel advances in sodium-based and ionic therapies add another layer: engineered sodium nanoparticles and salts are shown to selectively disrupt tumor homeostasis, exploit cancer’s aberrant sodium handling, and trigger immunogenic cell death [46]. For example, sodium chloride nanoparticles enhance intracellular sodium to toxic levels that drive both membrane rupture and immunogenic signaling, doubling as in situ anti-tumor vaccines when used in animal models [46]. Though the present study’s ES1–ES4 do not act via direct sodium overload, the principle that ionic species can play dual cytotoxic and immunomodulatory roles is strongly reinforced by emerging literature.

Recent studies on selenite and methylseleninic acid further highlight the versatility of salt/ionic anticancer agents: they act via induction of mitochondrial apoptosis, oxidative stress, and can also enhance chemosensitivity and engage multiple cell death pathways such as necrosis, autophagy, and ferroptosis [47]. Our results also suggest that while mitochondrial apoptosis is the primary route for ES1–ES4, additional mechanisms such as ER stress or necroptosis may be relevant and should be explored in future work.

Given that α,β-unsaturated enones are known Michael acceptors capable of reacting with nucleophilic thiol groups, the possibility of nonspecific electrophilic reactivity must be considered. However, the biological profile observed in the present study does not support indiscriminate cytotoxicity. The compounds exhibited a defined tumor–normal selectivity window and induced regulated apoptotic signaling events, including Bax/Bcl-2 modulation, caspase-3 activation, mitochondrial depolarization, and G2/M cell cycle arrest. Such coordinated, pathway-specific responses are more consistent with regulated apoptosis than with rapid, nonspecific chemical toxicity. Nevertheless, dedicated studies evaluating thiol reactivity or glutathione adduct formation would be required to fully dissect the contribution of intrinsic electrophilic reactivity, and this represents an important direction for future investigation.

Apoptosis-related assays were primarily performed at 48 h, as this time point corresponds to active execution of programmed cell death rather than secondary cytotoxic consequences observed at prolonged exposure. At 72 h, extensive loss of cell viability may reflect terminal stages of cell death, potentially confounding mechanistic interpretation. Therefore, 48 h was selected as the optimal window for mechanistic characterization.

### 3.4. Morphological and Cell Cycle Effects—G_2_/M Arrest and Context

Microscopic analysis confirmed a spectrum of morphological changes consistent with apoptosis, including membrane blebbing, cell shrinkage, and apoptotic bodies. In HCT-116 cells, these features were pronounced, consistent with observed quantitative sensitivity to ES1–ES4. Healthy MRC-5 cells, in contrast, remained primarily unaffected, both morphologically and in viability assays.

On the cell cycle level, ES1–ES4 impose significant G2/M phase arrest, as evidenced by increased G_2_/M populations (to ~44% in treated HCT-116 vs. 28% control). Arrest at G_2_/M is a frequently reported endpoint for enone-containing, alkylated, and partially ionic anticancer agents including, but not limited to, curcumin, dehydrozingerone, and silibinin derivatives [7,48,49]. The mechanistic basis is often attributed to DNA damage, altered energetics, or checkpoint activation, as demonstrated by SB-induced Drp1 mitochondrial fission and induced Cdc25C/p53 modulation. Importantly, robust G_2_/M arrest is clinically relevant, since it impedes mitosis and can sensitize tumor cells to DNA-damaging agents or radiation [50,51,52].

### 3.5. Salt Formation: Broader Implications for Drug Development

From a pharmaceutical sciences perspective, salt formation remains one of the most versatile methods for improving drug solubility, stability, dissolution rate, and overall developability, so much so that the majority of approved molecules are administered as salts [25,26]. New research continues to validate these advantages even for complex scaffolds and in nanoformulations that target localized delivery while reducing systemic toxicity [53]. Notably, sodium-based salts (as in ES1–ES4) offer the dual benefit of benign end-products and facile large-scale synthesis, key considerations for translational drug candidates.

Emerging work also demonstrates that the biochemical actions of salt/ionic drugs can be leveraged for synergistic chemo-immunotherapies; for instance, sodium chloride nanoparticles not only kill tumor cells but also promote anti-tumor immune responses by releasing danger signals [28]. This positions salt-modified molecules like ES1–ES4 as candidate agents for further preclinical evaluation, including assessment in combination strategies with established therapeutic modalities.

### 3.6. Study Limitations and Research Outlook

While the presented findings provide coherent cytotoxic and mechanistic evidence, several limitations must be acknowledged. Dedicated analytical purity profiling (e.g., NMR, IR, or HPLC analysis of the sodium salts) was not performed in the present study. All experiments were conducted in conventional two-dimensional in vitro models, which do not fully recapitulate tumor microenvironment complexity, three-dimensional tissue architecture, intercellular interactions, or pharmacokinetic constraints present in vivo. Although mitochondrial apoptosis and G2/M cell cycle arrest were identified as dominant mechanisms, future investigations should expand mechanistic profiling to include additional regulated cell death pathways such as necroptosis, autophagy, and ferroptosis, given their increasing relevance in anticancer therapy [54]. Furthermore, comprehensive toxicity profiling—particularly organ-specific safety evaluation (e.g., hepatic and cardiac models)—as well as direct comparative studies against clinically approved chemotherapeutics remain necessary. Validation in advanced three-dimensional tumor spheroid systems and in vivo efficacy models (including xenograft or orthotopic tumor models) will therefore represent critical next steps to establish translational relevance and therapeutic potential.

## 4. Materials and Methods

### 4.1. Preparation of Sodium Salts of α,β-Unsaturated Enones (ES1–ES4)

The parent enones (E1–E4), bearing a carboxylic acid group, were synthesized and fully characterized in our previous work (ref. [11]). The corresponding sodium carboxylates (ES1–ES4) were obtained by equimolar neutralization of the carboxylic acid function with aqueous NaOH (1.0 equiv) in ethanol at room temperature. After solvent removal under reduced pressure, the resulting sodium salts were dried and directly used in subsequent biological assays. As the transformation involves simple deprotonation of the carboxylic acid group without modification of the α,β-unsaturated enone scaffold, no structural rearrangement is expected. The salts were used without further purification.

### 4.2. Cell Lines, Chemicals, and Reagents

The cytotoxic potential of the test compounds was evaluated using two human cancer cell lines: colon adenocarcinoma (HCT-116, ATCC^®^ CCL-247™) and cervical carcinoma (HeLa, ATCC^®^ CCL-2™). To assess selectivity toward malignant cells, a normal human lung fibroblast cell line (MRC-5, ATCC^®^ CCL-171™) was included as a control. All cell lines were obtained from the American Type Culture Collection (ATCC, Manassas, VA, USA) and cultured under standard conditions at 37 °C in a humidified atmosphere with 5% CO_2_. Cells were maintained in complete growth medium consisting of Dulbecco’s Modified Eagle’s Medium (DMEM; Sigma-Aldrich, St. Louis, MO, USA, D5671), supplemented with 10% fetal bovine serum (FBS; Sigma-Aldrich, F7524), 1% non-essential amino acids (Sigma-Aldrich, M7145), 100 U/mL penicillin, and 100 μg/mL streptomycin (Roche Diagnostics GmbH, Mannheim, Germany, 11074440001). Medium was refreshed every 2–3 days, and cells were subcultured upon reaching 70–80% confluence. The sodium salts of α,β-unsaturated enones (ES1–ES4) were synthesized in-house (synthesis details are provided in Section 4.1). Stock solutions of the test compounds were prepared using the same solvent system as described for the parent enones in our previous study [11]. They were diluted in culture medium to the desired final concentrations immediately prior to treatment. Cisplatin (CAS 15663-27-1) was purchased from Sigma-Aldrich (St. Louis, MO, USA) and used as a positive control in all cytotoxicity assays.

### 4.3. Cytotoxicity Assessment by MTT Assay

The cytotoxic effects of sodium salts of α,β-unsaturated enones (ES1–ES4) were evaluated in vitro using the MTT assay in human colon cancer (HCT-116), cervical cancer (HeLa), and normal lung fibroblast (MRC-5) cell lines. Cells were seeded at a density of 5 × 10^3^ cells per well into 96-well plates and allowed to adhere overnight. Following attachment, cells were treated with serial dilutions of test compounds at final concentrations of 1, 3, 10, 30, and 100 μM. Treatments were carried out in triplicate and incubated under standard culture conditions (37 °C, 5% CO_2_) for 48 and 72 h.

After the incubation period, the culture medium was removed and replaced with 100 μL of MTT solution (5 mg/mL in PBS). Cells were further incubated for 3 h at 37 °C to allow for formazan formation. The resulting crystals were solubilized with 100 μL of pure dimethyl sulfoxide (DMSO; Merck, Darmstadt, Germany, CAS 67-68-5), and absorbance was measured at 595 nm using a microplate reader (Zenith 3100, Anthos Labtec Instruments GmbH, Salzburg, Austria).

Cell viability was calculated relative to untreated controls, and cytotoxicity (%) was expressed using the following formula:

Cytotoxicity (%) = [1 − (Absorbance of treated cells/Absorbance of control cells)] × 100.

Half-maximal inhibitory concentrations (IC_50_) were determined using nonlinear regression analysis in GraphPad Prism v10 (GraphPad Software, San Diego, CA, USA; https://www.graphpad.com). Selectivity index (SI) was calculated as the ratio of IC_50_ in MRC-5 fibroblasts to IC_50_ in the respective cancer cell line [22] using the following formula:

SI = IC_50_ (MRC-5)/IC_50_ (cancer cells)

An SI value greater than 1 indicates selective cytotoxicity toward tumor cells.

### 4.4. Analysis of Cell Morphology

To evaluate treatment-induced morphological changes, HCT-116, HeLa, and MRC-5 cells were seeded in 24-well plates and treated with various concentrations of sodium salts of α,β-unsaturated enones (ES1–ES4) for 48 and 72 h under standard culture conditions. Untreated control cells were maintained in parallel for each time point.

Following incubation, cellular morphology was assessed using phase-contrast microscopy at 100× magnification (Olympus IX50 inverted microscope, Olympus Corporation, Tokyo, Japan). Changes in cell appearance were documented and evaluated for features indicative of cytotoxic response, such as cell shrinkage, rounding, detachment, and reduced adherence [22].

Images were analyzed using ImageJ software version 1.54 (National Institutes of Health, Bethesda, MD, USA; https://imagej.net/ij/.

### 4.5. Assessment of Apoptosis by Annexin V-FITC/7-AAD Staining

The mode of cell death induced by sodium salts of α,β-unsaturated enones (ES1–ES4) was evaluated in HCT-116 and HeLa cell lines using Annexin V-FITC/7-AAD staining and flow cytometry. Cells were seeded into 24-well plates at a density of 1 × 10^5^ cells per well and treated with the respective IC_50_ concentrations of each compound. After 48 and 72 h of incubation, cells were harvested, washed twice with cold phosphate-buffered saline (1× PBS, without Ca^2+^/Mg^2+^), and resuspended in 100 μL of ice-cold 1× binding buffer. Apoptotic staining was performed by adding 10 μL of Annexin V-FITC and 20 μL of 7-AAD (FITC Annexin V Apoptosis Detection Kit with 7-AAD, BioLegend, San Diego, CA, USA, Cat. No. 640922) to each sample. The suspensions were incubated in the dark for 15 min at 4 °C. Following staining, 400 μL of binding buffer was added, and samples were immediately analyzed using a Cytomics FC500 flow cytometer (Beckman Coulter, Brea, CA, USA).

Data acquisition and analysis were performed using FlowJo software v10.6 (BD Biosciences, Franklin Lakes, NJ, USA). Results were expressed as dot plots distinguishing early apoptotic, late apoptotic, necrotic, and viable cell populations [55].

### 4.6. Flow Cytometric Analysis of Apoptosis-Related Proteins

To investigate the modulation of apoptosis-related signaling, HCT-116 and HeLa cells were treated with IC_50_ concentrations of sodium salts of α, β-unsaturated enones (ES1–ES4) for 48 h. Untreated cells maintained in complete medium served as controls.

Following treatment, cells were harvested, washed twice with phosphate-buffered saline (PBS), and processed using the Fixation and Permeabilization Kit (eBioscience, Thermo Fisher Scientific, Waltham, MA, USA) according to the manufacturer’s instructions. Fixed and permeabilized cells were then stained with primary antibodies specific to pro- and anti-apoptotic markers: mouse anti-human Bcl-2 (clone DC21, sc-783, Santa Cruz Biotechnology, Dallas, TX, USA), mouse anti-human Bax (clone N20, sc-493, Santa Cruz Biotechnology, Dallas, TX, USA), and anti-cleaved caspase-3 (Cell Signaling Technology, Danvers, MA, USA, 9661). After primary incubation, cells were washed and subsequently incubated for 30 min with Alexa Fluor^®^ 488-conjugated secondary antibodies (ab185015, Abcam, Cambridge, UK), also following the manufacturer’s instructions.

After final washing with PBS, samples were analyzed by flow cytometry using a Cytomics FC500 flow cytometer (Beckman Coulter, Brea, CA, USA). Data were processed using FlowJo software (BD Biosciences, Franklin Lakes, NJ, USA).

Protein expression levels were quantified based on mean fluorescence intensity (MFI). Results were presented as bar graphs for Bcl-2 and Bax, and the Bax/Bcl-2 ratio was calculated as:

Bax/Bcl-2 index = Bax MFI/Bcl-2 MFI.

The percentage of cleaved caspase-3-positive cells was evaluated and visualized in histogram plots [22,55].

### 4.7. Cell Cycle Analysis

Cell cycle distribution was evaluated in HCT-116 and HeLa cells following treatment with IC_50_ concentrations of sodium salts of α,β-unsaturated enones (ES1–ES4). Untreated cells cultured under identical conditions served as controls.

After 48 h of treatment, cells were harvested, washed twice with phosphate-buffered saline (PBS), and fixed in 70% ice-cold ethanol at 4 °C overnight. The next day, cells were pelleted by centrifugation and resuspended in 1 mL PBS containing RNase A (Thermo Fisher Scientific, Waltham, MA, USA) at a final concentration recommended by the manufacturer. Samples were incubated at 37 °C for 30 min to eliminate residual RNA.

Following RNase treatment, cells were stained with propidium iodide (1 μg/mL in PBS; Sigma-Aldrich, St. Louis, MO, USA) and incubated in the dark for 15 min at room temperature. Flow cytometric analysis was performed using a Cytomics FC500 flow cytometer (Beckman Coulter, Brea, CA, USA).

DNA content was quantified, and cell cycle phase distribution (G_0_/G_1_, S, and G_2_/M) was determined using Flowing Software v2.5.2 (Flowing Software, Perttu Terho, Turku, Finland). Results were expressed as histograms.

### 4.8. Determination of Cytochrome c Expression and Localization by Immunofluorescence

To assess the intracellular localization and expression of cytochrome c, immunofluorescence analysis was performed in HCT-116 and HeLa cells following treatment with IC_50_ concentrations of sodium salts of α,β-unsaturated enones (ES1–ES4) for 48 h.

Cells were cultured on sterile glass coverslips and, after treatment, washed with phosphate-buffered saline (PBS) and fixed with 4% formaldehyde for 20 min at room temperature. Fixed cells were permeabilized using 0.2% Tween-20 and subsequently blocked with a buffer containing 0.1% Tween-20 and 10% fetal bovine serum (FBS) for 30 min.

Immunolabeling was performed using a monoclonal mouse anti-human cytochrome c antibody (1:100 dilution; G7421, Promega Corporation, Madison, WI, USA), incubated for 1 h at room temperature. After washing, cells were incubated for 30 min with a fluorescein isothiocyanate (FITC)-conjugated goat anti-mouse secondary antibody (1:200 dilution in blocking buffer). Following final washes with PBS, coverslips were mounted, and fluorescence imaging was performed using an inverted fluorescence microscope (Olympus IX50) [56].

Image analysis and quantification of cytochrome c distribution were carried out using ImageJ software (National Institutes of Health, Bethesda, MD, USA).

### 4.9. Determination of Mitochondrial Membrane Potential (ΔΨm) by JC-10 Analysis

Mitochondrial membrane potential (ΔΨm) was assessed in HCT-116 and HeLa cells after 48 h treatment with IC_50_ concentrations of sodium salts of α,β-unsaturated enones (ES1–ES4) using the JC-10 dye (Enzo Life Sciences, Farmingdale, NY, USA), as previously described [56].

JC-10 is a dual-emission fluorescent dye that selectively accumulates in mitochondria. In cells with intact mitochondrial potential, the dye aggregates and emits red fluorescence. Upon depolarization, the dye remains in its monomeric form in the cytosol, emitting green fluorescence. The shift from red to green emission serves as an indicator of mitochondrial dysfunction.

Cells were seeded in 24-well plates at a density of 3 × 10^4^ cells per well and incubated overnight at 37 °C in a humidified atmosphere with 5% CO_2_. The next day, cells were treated with the test compounds for 48 h. Following treatment, cells were washed with pre-warmed 1× PBS and incubated with JC-10 dye solution (2.5 μM in warm PBS) for 20 min at 37 °C under 5% CO_2_.

After incubation, cells were washed again with 1× PBS and immediately analyzed using an Olympus IX50 inverted fluorescence microscope. Fluorescence images were acquired, and the ratio of green (525 nm) to red (590 nm) fluorescence intensity was calculated using ImageJ software (NIH, Bethesda, MD, USA) to quantify changes in mitochondrial membrane potential.

### 4.10. Statistical Analysis

All experiments were performed in three independent biological replicates. Experimental data were analyzed using SPSS Statistics software, version 20 (IBM Corp., Armonk, NY, USA). The normality of data distribution was assessed using appropriate tests based on sample size. For normally distributed data, comparisons between each treated group and the corresponding control group were performed using the independent samples Student’s *t*-test. For non-normally distributed data, the nonparametric Mann–Whitney U test was applied. Results are presented as mean ± standard deviation (SD). Statistical significance was considered at *p* < 0.05 (*) and *p* < 0.01 (**).

## 5. Conclusions

In conclusion, vanillin-derived α,β-unsaturated enone sodium salts (ES1–ES4) exhibit time-dependent and selective cytotoxic activity against colorectal (HCT-116) and cervical (HeLa) cancer cells while maintaining lower toxicity toward normal fibroblasts (MRC-5) under in vitro conditions. Direct comparison with the previously reported parent enones (E1–E4) [11] indicates that salt formation does not enhance intrinsic cytotoxic potency; however, selective anticancer activity and a consistent mechanistic profile are preserved. ES1–ES4 induce mitochondrial-mediated apoptosis, as evidenced by Bax/Bcl-2 modulation, caspase-3 activation, mitochondrial membrane depolarization, cytochrome c release, and G2/M cell cycle arrest. While these findings provide mechanistic insight into the biological behavior of sodium enone salts, further validation in advanced preclinical models will be necessary to determine their translational relevance.

## Figures and Tables

**Table 1:** IC_50_ values (μM) and selectivity index (SI) of enone sodium salts (ES1–ES4) and cisplatin in cancer and normal cell lines after 48 and 72 h of treatment.

Compound	IC_50_ (µM)							
	48 h			72 h			SI 72 h	
	HeLa	HCT-116	MRC-5	HeLa	HCT-116	MRC-5	HeLa	HCT-116
ES1	51.3 ± 0.2	32.7 ± 1.8	>100	20.7 ± 1.1	14.9 ± 0.8	88.9 ± 1.3	4.30	5.98
ES2	49.8 ± 1.2	30.9 ± 2.1	>90	18.1 ± 0.9	14.3 ± 0.8	91.3 ± 1.3	5.04	6.41
ES3	54.7 ± 2.0	55.3 ± 2.1	>100	23.1 ± 1.8	14.9 ± 0.9	76.4 ± 1.4	3.30	5.12
ES4	55.2 ± 1.9	77.1 ± 3.8	>100	29.1 ± 1.4	15.7 ± 1.0	84.5 ± 1.2	2.91	5.40
CisPt	16.5 ± 1.0	13.6 ± 1.5	31.0 ± 2.7	3.6 ± 0.8	7.1 ± 1.6	32.1 ± 1.1	8.87	4.53

## Data Availability

The data presented in this study are available in this article.
